# Mapping of Signaling Pathways Linked to sIgAD Reveals Impaired IL-21 Driven STAT3 B-Cell Activation

**DOI:** 10.3389/fimmu.2019.00403

**Published:** 2019-03-18

**Authors:** Andri L. Lemarquis, Fannar Theodors, Helga K. Einarsdottir, Bjorn R. Ludviksson

**Affiliations:** ^1^Department of Immunology, Landspítali–The National University Hospital of Iceland, Reykjavík, Iceland; ^2^Faculty of Medicine, University of Iceland, Reykjavík, Iceland

**Keywords:** selective IgA deficiency, IL-21, pSTAT3, phosphoflow, B cells, T cells, IgA

## Abstract

**Objectives:** It has recently been shown that individuals with selective IgA deficiency (sIgAD) have defective B cell responses both to T cell dependent and independent mimicking stimulations. The complex intracellular signaling pathways from different stimuli leading to IgA isotype switching have not been fully elucidated. Thus, the main objective of this study was to delineate these pathways and their potential role in the immunopathology linked to sIgAD.

**Materials and Methods:** PBMCs from 10 individuals with sIgAD and 10 healthy controls (HC) were activated *in vitro* via either a T cell dependent or independent mimicking stimulation. Intracellular phosphorylation of pSTAT3, pSTAT5, pSTAT6, and as pERK1/2 was evaluated in T and B cells using phosphoflow cytometry.

**Results:** By evaluating T cell dependent cytokine driven pathways linked to IgA isotype induction we identified a defect involving an IL-21 driven STAT3 activation isolated to B cells in sIgAD individuals. However, all other signaling pathways studied were found to be normal compared to HC. In T cell dependent cytokine driven stimulations linked to IgA isotype induction the following patterns emerged: (i) IL-10 led to significant STAT3 activation in both T- and B cells; (ii) IL-4 stimulation was predominantly confined to STAT6 activation in both T- and B cells, with some effects on STAT3 activation in T-cells; (iii) as expected, of tested stimuli, IL-2 alone activated STAT5 and some STAT3 activation though in both cases only in T-cells; (iv) IL-21 induced significant activation of STAT3 in both T- and B cells, with some effects on STAT5 activation in T-cells; and finally (v) synergistic effects were noted of IL-4+IL-10 on STAT5 activation in T-cells, and possibly STAT6 in both T- and B cells. On the other hand, CPG induced T cell independent activation was confined to ERK1/2 activation in B cells.

**Conclusion:** Our results indicate a diminished STAT3 phosphorylation following IL-21 stimulation solely in B cells from sIgAD individuals. This can represent aberrant germinal center reactions or developmental halt. Thus, our work provides further insight into the unraveling of the previously hypothesized role of IL-21 to reconstitute immunoglobulin production in primary antibody deficiencies.

## Introduction

Selective IgA deficiency (sIgAD) is the most common primary antibody deficiency (PAD) in Caucasians characterized by an increased risk of autoimmunity, atopic diseases, and infections (1). It is defined as serum IgA ≤ 0.07 g/L with normal serum levels of IgG and IgM (2). In the normal host multiple factors are known to be needed for a correct B cell maturation to IgA secretion. These include the use of correct genetic information, transcription of key molecules and complex intracellular signaling pathways (3). Even though genes encoding for IgA have not been shown to be abnormal in sIgAD its production remains defective (4). Aberrations are seen in early maturing B cell lineages (5–7) and the defect has been corrected through stem cell transplant by correcting a defect in B cell ontogeny (8). B cells from sIgAD individuals have been shown to be able to produce little amounts of IgA after various T cell dependent and independent mimicking *in vitro* stimulations. Most commonly this includes CD40L with TGF-ß1, IL-2, IL-4, IL-10, and IL-21 at various concentrations and combinations (5, 6, 9–12). However, despite successful IgA secretion in these T-cell dependent stimulatory conditions, they have not been able to rectify IgA levels up to normal compared to healthy controls (5, 6, 9–12). Some have hypothesized that this could be used in the treatment of hypogammaglobulinemia but the problem is that such stimulation has been shown to lead to faulty longevity (7). TLR9 is known to be a strong inducer of IgA secretion in healthy individuals but were recently shown to be defective in sIgAD (7). Given the importance of TLR9 at mucosal surfaces and its potential defect in sIgAD, studying this receptor might provide new insights in its connection to IgA secretion and mucosal immunology (7).

JAK-STAT signaling is known to be essential in the intracellular transduction following activation of cells by common gamma chain cytokines, IL-2, IL-4, IL-7, IL-9, IL-15, and IL-21; all known to affect class switching to IgA, and its subsequent production by B cells (13). Of these IL-21 has been of special interest and often theorized as having the strongest therapeutic potential in sIgAD (3, 5). Interestingly while many of the common gamma chain cytokines are known to have impaired IgA inducing capacities in sIgAD they have been shown to use similar intracellular signaling cascades (14). Complete gain of function and loss of function in the JAK-STAT pathways have been shown to lead to severe phenotypes with lymphocyte affections (15). Possibly a slighter defect or partial affection may lead to different phenotypes and makes it essential to further elucidate them and their potential role in this disease. Especially since the cytokines known to be defective in sIgAD are described as using similar signaling cascades. But to do so one needs to assess the reactivity of specific populations to stimuli it is not always well-defined how these may affect different pathways in different cell subgroups.

The development of novel biological targets in signaling pathways for the treatment of various diseases related to immune modulation (16) has awoken an interest in phosphorylation defects in immune mediated diseases (17). Signaling defects have furthermore been described in similar PIDs to sIgAD, like common variable immune deficiency (CVID), where a concomitant risk for the development of autoimmunity is known (18). CVID individuals have been shown to have both a CpG-driven STAT3 (19) and a CD40L and αIgM driven p-AKT B cell defects (18).

Thus, it is important to understand the intracellular responsiveness of cytokines inducing IgA production and evaluate the responses in sIgAD B- and T cells. That way it may be possible to elucidate if the defects in IgA production are related to altered signaling as in CVID. The main objective of this study was therefore to investigate intracellular pathways in B and T cells of sIgAD individuals and healthy controls (HCs) by examining phosphorylation responses using multicolor flow cytometric phosphoflow analysis.

## Materials and Methods

### sIgAD Patients and Healthy Donors

Peripheral blood from 10 individuals from the Icelandic sIgAD cohort (1) were collected into heparinized tubes after obtaining informed consent (in accordance with procedures approved by The National Bioethics Committee and The Data Protection Authority in Iceland). These were compared to gender matched healthy controls from the blood bank. None of the patients had ongoing acute infection at the time of sample collection or were receiving immunosuppressive therapy. The adult sIgAD diagnosis was based on the criteria from ESID Registry, which is an individual over 17 years of age with a serum IgA of <7 mg/dl (0.07 g/L) but normal serum IgG and IgM, in whom other causes of hypogammaglobinaemia had been excluded (2, 20).

### Isolation of Peripheral Blood Mononuclear Cells

Peripheral Blood Mononuclear cells (PBMCs) were isolated from heparinized peripheral blood using Ficoll–Paque gradient centrifugation (Sigma-Aldrich, St. Louis, MO, USA).

### Sample Freezing and Storage

After isolation the cells were suspended in X-vivo 20 serum free medium (Lonza; Walkersville, Maryland, USA). Cells frozen in X-vivo were supplemented with ProFreeze chemically defined freeze medium (Lonza; Verviers, Belgium). Cell suspensions were mixed with DMSO (Sigma Aldrich) to prevent damage and then cryopreserved in liquid nitrogen.

### Phospho Protein Staining for Validation of Protocol

A phosphoflow stimulation and staining protocol was established and optimized which was based on methods previously published (21, 22). Briefly; cryotubes were taken out of liquid nitrogen storage and thawed quickly in a 37°C water bath. They were then gently pipetted using Pasteur pipettes into 50 ml conical tube containing 25 ml of IMDM medium. Cells were then centrifuged at room temperature at 300xg for 10 min and the supernatant discarded. Washing was repeated with 25 ml of IMDM medium and the cell pellet was re-suspended in medium and counted. 5 × 10^5^ cells per well were aliquoted into sterile 96 well plates (#163320 Thermo fisher, Waltham, Massachusetts, USA). The cells were then incubated for 1 h at 37°C in a 5% CO_2_ incubator to let the cells reach a basal phosphorylation. Cells were stimulated with various concentrations and combinations of cytokines for 15 min at 37°C and then fixed in 1.8% formaldehyde for 10 min at room temperature. Cells were then pelleted and centrifuged at 1600 RPM at 4°C for 10 min. Supernatants were then discarded, and cells washed with 200 μl of staining buffer. Cells were then permeabilized with 150 μl of ice-cold 95% methanol. This was followed by washes, first with 80 μl of staining buffer and then repeated with 200 μl of staining buffer. Cell pellet was re-suspended in the residual volume and 50 μl of that was transferred to fresh 96-well plate. Staining with fluor conjugated antibodies was carried out in the dark for 30 min at room temperature. One more wash was performed with 100 μl of staining buffer and then the samples were put on ice until analysis with Beckman Coulter Navios flow cytometer. For each analysis a minimum of 50.000 events were collected into a defined lymphocyte gate. Antibodies used were Alexa flour 488 anti-human ERK1/2 (T202/Y204) (#53-9109-42 Ebioscience, Vienna, Austria), PE anti-human pSTAT1(Y701) (#12-9008-42 Ebioscience), APC anti-human pSTAT3 (Y705) (#17-9033-42 Ebioscience), PE-Cyanine 7 anti-human pSTAT5 (Y694) (#25-9010-42 Ebioscience), Pacific Blue anti-human p38 (T180/Y182) (#560313 BD biosciences, San Jose California, USA), PerCp Cy 5.5 anti human CD20 clone H1 cytoplasmic tail (#55802 1 BD biosciences), Brilliant violet 510 anti-human CD3 (clone UCHT1 # 300448 Biolegend, San Diego California, USA). Stimulations used were IL-2 (50 ng/ml), IL4 (100 ng/ml), IL-10 (100 ng/ml), IL-21(50 ng/ml) (all from R&D systems, Minneapolis Minnesota, USA), and CpG-ODN2006 (1 μg/ml TLR9 agonist, InvivoGen, San Diego California, USA).

### Flow Cytometric Analysis

Flow cytometric data was analyzed with Kaluza version 1.3. Sample analysis was performed in a blinded manner, which is to say that the researcher could not know which group a sample belonged to until after the analysis was complete. When testing activation induced phosphorylation, results are displayed as fold change of the GMFI (geometric mean of fluorescence intensity) value of the stimulated vs. unstimulated sample. Examples of phosphorylation and gating strategies to be seen in Supplement Figures 2,3.

### Data Presentation and Statistical Analysis

Graphical data presentation and Statistical analysis was performed with Graph Pad Prism version 8.0.1. To test for normal distribution of data Shapiro-Wilk test was performed. Datasets did not all pass normality tests and therefore Kruskal–Wallis 1-way ANOVA was performed on datasets with Dunn's post-test.

## Results

### Mapping of T-Cell Dependent and Independent Intracellular Phosphorylation of STAT3, STAT5, STAT6, and ERK1/2 Related to IgA Induction Reveals a Complex and Heterogeneous Phosphorylation Pattern

**Figure 1:**
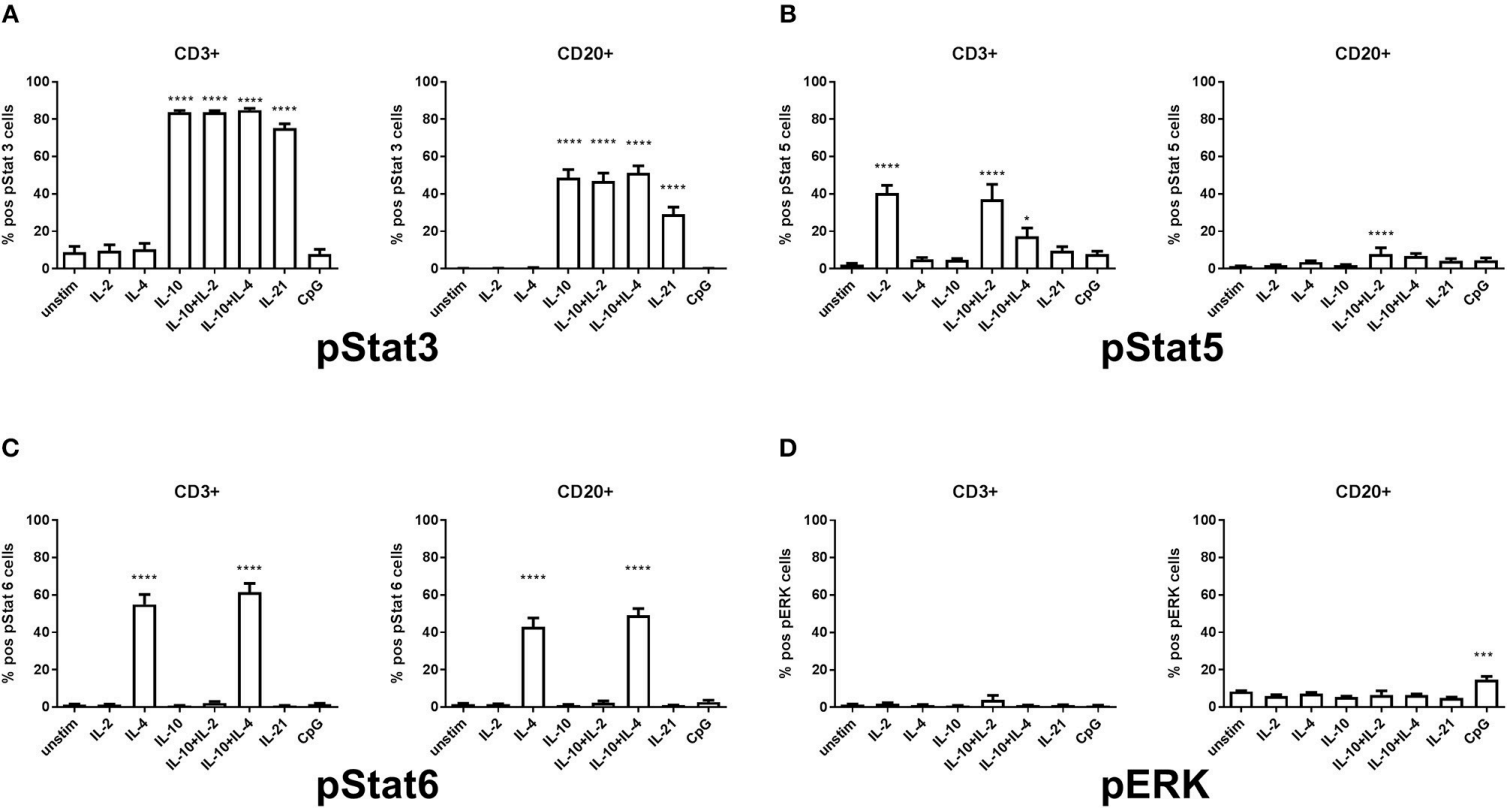
Differences in percentage of pSTAT3, pSTAT5, pSTAT6, and ERK1/2 expression in T and B cells in healthy controls. The figure shows the % of expression of pSTAT3 (S1A), pSTAT5 (S1B), pSTAT6 (S1C), and ERK1/2 (S1D) as measured *ex vivo* in PBMCs after 15min stimulation of IL-2 (50 ng/ml), IL4 (100 ng/ml), IL-10 (100 ng/ml), IL-21 (50 ng/ml), or CpG ODN 2006 (1 μg/ml TLR9 agonist) alone or in combinations in CD3 + T and CD20 + B cells of healthy individuals. **(A)** shows the percentage of pSTAT3 positive T (CD3+) and B cells (CD20+). A positive response is seen with IL-10, IL-10 + IL-2, IL-10 + IL-4, and IL-21 stimulation but none after IL-2, IL-4 alone, or CpG. **(B)** shows the percentage of pSTAT5 positive T (CD3+) and B cells (CD20+). A positive response is seen with IL-2, IL-10 + IL-2, and IL-10 + IL-4 in T cells but IL-10 + IL-2 in B cells, but no activation in neither T nor B cells after IL-4, IL-10, IL-21, or CpG. **(C)** shows the percentage of pSTAT6 positive T (CD3+) and B cells (CD20+). A positive response is seen with IL-4 and IL-10 + IL-4 in T and B cells, but no activation in neither T nor B cells after IL-2, IL-10, IL-10 + IL-2, IL-21, or CpG. **(D)** shows the percentage of ERK1/2 positive T (CD3+) and B cells (CD20+). A positive response is seen with CpG stimulation in B cells but not in T cells. Furthermore, no activation is seen in either T nor B cells after IL-2, IL+4, IL-10, IL-10 + IL-2, IL-10 + IL-4, IL-21, or CpG. Significance was calculated in relation to the control group. ***p < 0.001, ****p < 0.0001 (as determined by one-way ANOVA).

**Figure 2:**
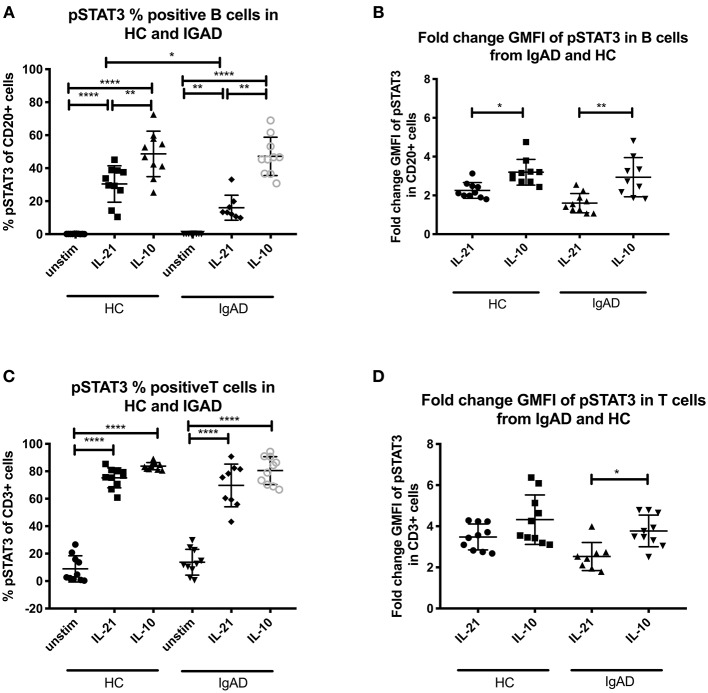
The percentage and fold change GMFI of pSTAT3 in B (CD20+) and T cells (CD3+) after IL-21 and IL-10 in sIgAD individuals and healthy controls. The figure shows % of phosphorylated and fold change GMFI of STAT3 as measured *ex vivo* in PBMCs after 15min stimulation of IL-21 (50 ng/ml), IL-10 (100 ng/ml) + IL4 (100 ng/ml) and IL-2 (50 ng/ml). Analysis of **(A)** % pSTAT3 positive B cells (CD20+) and **(B)** fold change GMFI shows a significant lower percentage of B cells expressing pSTAT3 after IL-21 stimulation but no differences between phosphorylation responses after IL10 in sIgAD and HCs. Analysis of **(C)** % pSTAT3 positive T cells (CD3+) and **(D)** fold change GMFI shows no differences between phosphorylation responses after IL-21 and in sIgAD compared to HCs. Significance was calculated in relation to the control group. *p < 0.05, **p < 0.01, ****p < 0.0001 (as determined by one-way ANOVA).

Lymphocytes from HCs demonstrate a variable pattern of pSTAT3, pSTAT5, pSTAT6, and ERK1/2 depending on the given underlying stimulatory conditions (Figure 1). In our model it was important to assess the stimulations previously shown in the literature to be defective in sIgAD, that is IL-2, IL-4, IL-10, IL-21, and CpG. Furthermore, it was important to try the concomitant stimulation of IL-10 and either IL-2 and/or IL-4 since it has been shown that these improve IgA secretion in sIgAD (10). In exploring the responses of these above-mentioned stimuli in pSTAT3 expression a good response was seen after IL-10 and IL-21 separately in both T and B cells (Figure 1A). However, neither antagonistic nor synergistic effects were found in any of the conditions tested (Figures 1A, 2A,C). The mapping of STAT5 signaling following various T-cell dependent and independent mimicking stimulations revealed that significant phosphorylation was confined to T-cells. Of the stimulations tested, IL-2 alone induced phosphorylation in T cells. Dual stimulation with IL-2 and IL-10 induced phosphorylation in both T- and B cells. Dual stimulation with IL-4 and IL-10 did also induce significant STAT5 phosphorylation in T cells but not B cells while alone neither IL-4 nor IL-10 alone did induce significant STAT5 phosphorylation in B cells. Regarding pSTAT6 expression, expression was seen after IL-4 stimulation in both T- and B cells (Figure 1C). For T cell independent responses, the evaluation ERK1/2 expression was done following CPG stimulation and where expression was seen following stimulation in a small proportion of B cells in both HCs and IgAD (**Figure 5A**, HC B cells 17.7% +/– 1.0 vs. sIgAD B cells 13.8% +/– 2.6).

### IL-21 Driven Phosphorylation of STAT3 Is Lower in sIgAD B Cells Compared to HCs

Since when exploring pSTAT3 expression after various stimuli a good response was seen after IL-10 and IL-21 separately (Figure 1A) these where tested in sIgAD. Proportionally fewer B cells responded to IL-21 driven STAT3 phosphorylation in sIgAD compared to HCs (Fraction of HC B cells = 30.4% +/−11.5 vs. sIgAD B cells 16.1% +/– 7.5; *p* = 0.03; Figures 2A,C) but not in GMFI fold change (HC B cells = 2.3+/−0.3 vs. sIgAD B cells = 1.6 +/– 0.3; *p* < 0.14; Figures 2B,D). In addition, when evaluating non-fold change adjusted GMFI for the pSTAT3 activation the sIgAD demonstrated a lower response (HC vs. sIgAD pSTAT3 GMFI 4.74 vs. 3.61; *p* = 0.032), and the fold GMFI change difference tended also to be lower for the sIgAD group compared to HCs (HC vs. sIgAD pSTAT3 fold change GMFI 2.26 vs. 1.60; *p* = 0.098).

### STAT5 Phosphorylation Is Seen in After IL-2 and Concomitant IL-4 With IL-10 Stimulation

**Figure 3:**
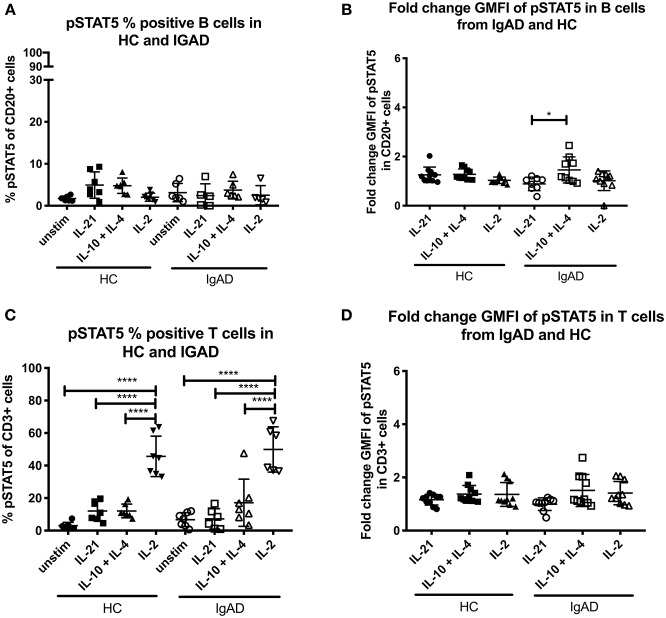
The percentage and fold change GMFI of pSTAT5 in B (CD20+) and T cells (CD3+) after IL-21, IL-10 + IL4, and IL-2 in sIgAD individuals and healthy controls. The figure shows % of phosphorylated and fold change GMFI of STAT5 as measured *ex vivo* in PBMCs after 15min stimulation of IL-21 (50 ng/ml), IL-10 (100 ng/ml) + IL4 (100 ng/ml), and IL-2 (50 ng/ml). Analysis of **(A)** % pSTAT5 positive B cells (CD20+) and **(B)** fold change GMFI shows a no significant difference in the percentage or fold change GMFI of B cells expressing pSTAT5 after IL-21, IL-10 + IL4, and IL-2 stimulation in sIgAD and HCs. Analysis of **(C)** % pSTAT5 positive T cells (CD3+) and **(D)** fold change GMFI shows neither differences between phosphorylation responses in T cells after IL-21, IL-10 + IL4, and IL-2 stimulation in sIgAD and HCs. Significance was calculated in relation to the control group. *p < 0.05, ****p < 0.0001 (as determined by one-way ANOVA).

STAT5 signaling in healthy controls revealed that significant phosphorylation after IL-2 stimulation was confined to T-cells (Figure 1B). In sIgAD it has been shown that the addition of IL-2 to IL-10 stimulation leads to higher “rescue production” of IgA after stimulation compared to IL-10 alone (10). In our setup a difference was seen in percentage of phosphorylation in STAT5 in B cells with IL-21, IL-10 + IL-4, or IL-2 alone for HCs (Figure 1B). When analyzing these stimulations in sIgAD compared to HCs we did not observe any differences between the two groups in cell proportions or fold change GMFI (Figures 3A–D) following IL-21, dual IL-10 with IL-4 or IL-2 stimulation.

### Only IL-4 Induces a Significant STAT6 Phosphorylation in Both T- and B Cells

**Figure 4:**
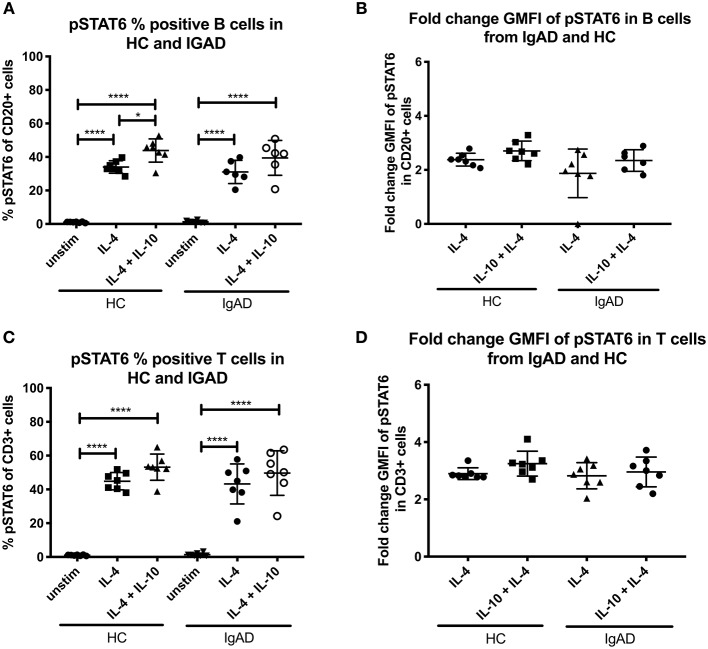
The percentage and fold change GMFI of pSTAT6 in B (CD20+) and T cells (CD3+) after IL-4 and IL-10 + IL4 in sIgAD individuals and healthy controls. The figure shows % of phosphorylated and fold change GMFI of STAT6 as measured *ex vivo* in PBMCs after 15-minute stimulation of IL-4 (100 ng/ml) and IL-10 (100 ng/ml) + IL4 (100 ng/ml). Analysis of **(A)** % pSTAT6 positive B cells (CD20+) and **(B)** fold change GMFI shows a no significant difference in the percentage or fold change GMFI of B cells expressing pSTAT6 after IL-4 or IL-10 + IL4 stimulation in sIgAD and HCs. Analysis of **(C)** % pSTAT6 positive T cells (CD3+) and **(D)** fold change GMFI shows neither differences between phosphorylation responses in T cells after IL-4 or IL-10 + IL4 stimulation in sIgAD and HCs. Significance was calculated in relation to the control group. *p < 0.05, ****p < 0.0001 (as determined by one-way ANOVA).

pSTAT6 expression was only seen after IL-4 stimulation in both T- and B cells, with slight but noticeable synergistic effects following dual IL-4 and IL-10 stimulations in B cells only (Figure 1C). No differences where seen in the fraction nor fold change GMFI between the two cohorts after IL-4 or IL-4 + IL-10 stimulation (Figures 4A–D).

### Minimal ERK Activation in B Cells Following CpG Stimulation

**Figure 5:**
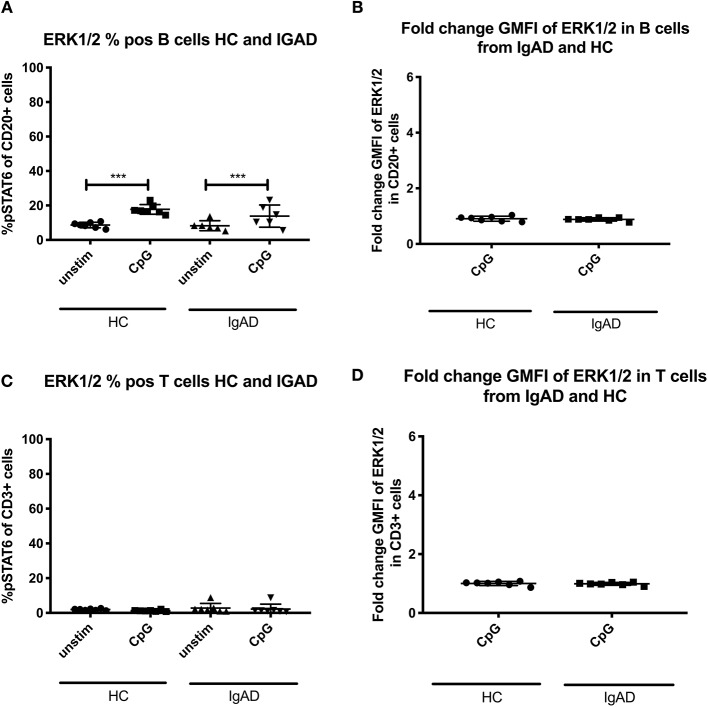
Effects of CpG on ERK1/2 expression in B and T cells from sIgAD individuals and healthy controls. The figure shows the % of expression and fold change GMFI of ERK1/2 as measured *ex vivo* in PBMCs after 15min stimulation of CpG ODN 2006 (1 μg/ml TLR9 agonist). Analysis of **(A)** % ERK1/2 positive B cells (CD20+) and **(B)** fold change GMFI shows a no significant difference in the percentage or fold change GMFI of B cells expressing ERK1/2 after CpG stimulation in sIgAD and HCs. Analysis of **(C)** % CpG positive T cells (CD3+) and **(D)** fold change GMFI shows neither differences between phosphorylation responses in T cells after CpG stimulation in sIgAD and HCs. Significance was calculated in relation to the control group. ***p < 0.001 (as determined by one-way ANOVA).

Since expression of ERK1/2 was only seen following T-cell independent stimulation in HCs and since its basal phosphorylation of ERK1/2 without stimulation (Supplement Figures 1A,B) did not differ significantly between T or B cells in both groups, sIgAD and HCs, it was hypothesized that some differences could be seen in sIgAD since stimulation with CpG leads to defective IgA production in sIgAD. No differences where seen in ERK1/2 expression in sIgAD compared to HCs in our model in T or B cells (Figures 5A,B).

### IL-21 Driven STAT3 Phosphorylation Is Diminished in sIgAD B Cells

**Figure 6:**
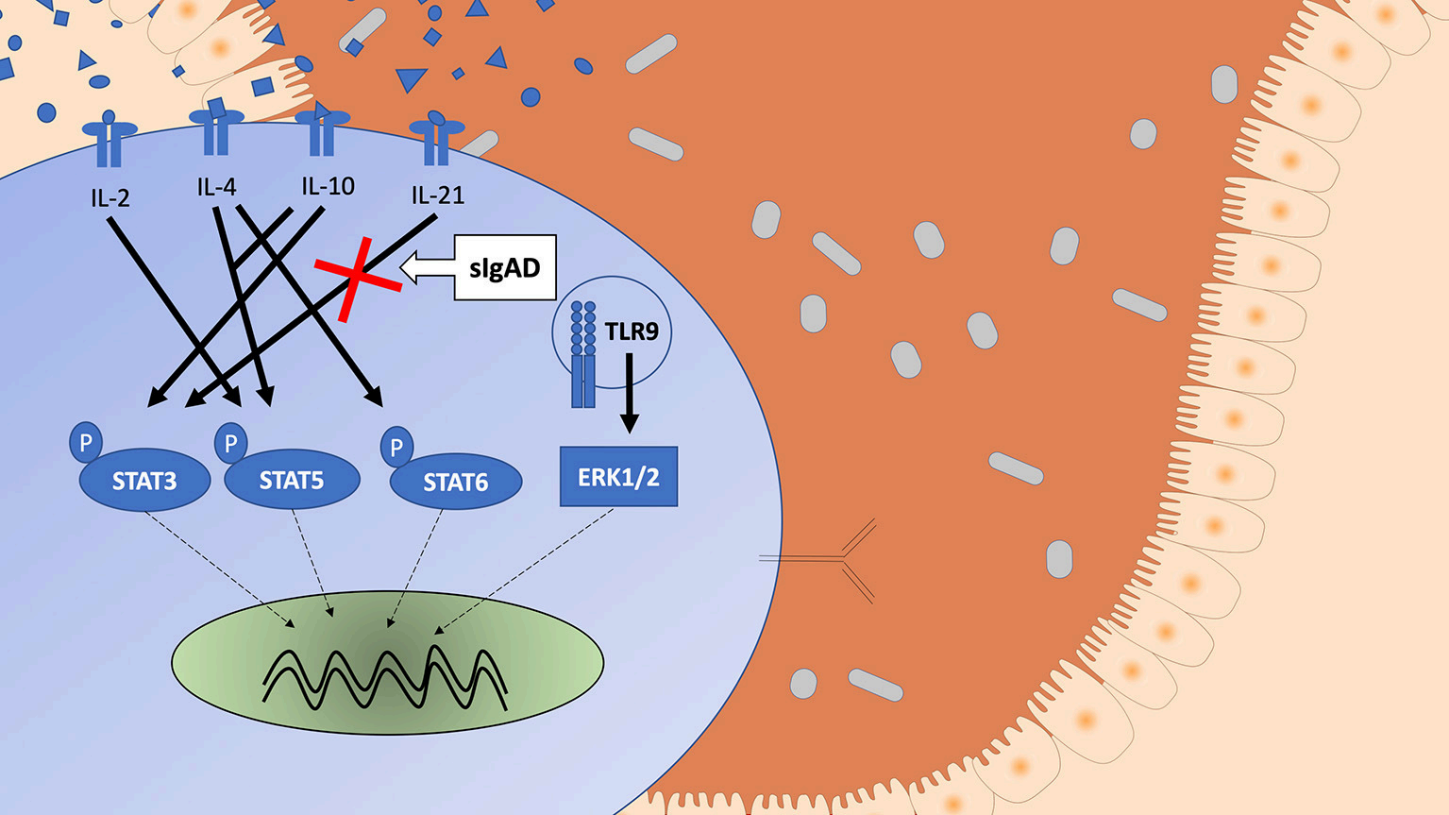
A schematic figure showing the intracellular phosphorylation responses in healthy individuals of STAT3, STAT5, STAT6, and ERK1/2 expression after stimulation with IL-2, IL4, IL-10 ± IL-2 or IL-4, IL-21, and CpG based on the results from Supplement Figure 1. The figure shows a visual representation of a B cell in IgAD and the different stimuli tested, differences are seen in activation based on stimuli. The Stimuli leading to activation are shown with an arrow with an unbroken line (→ ) and the potential signal leading to intranuclear translation with an arrow with a broken line (- - → ). STAT3 phosphorylation is seen after IL-10 and IL-21 stimulation. STAT5 phosphorylation is seen after IL-2 and IL-4 with IL-10 stimulation but with neither alone. STAT6 phosphorylation is seen after IL-4 stimulation. ERK1/2 expression is seen after TLR9 activation. A defect is seen in sIgAD in B cells in pSTAT3 after IL-21 stimulation.

Since STAT3 phosphorylation was highly upregulated following both IL-10 and IL-21 separately it was important to evaluate their role in sIgAD (Figure 2). Interestingly, as shown in Figure 2A, significantly fewer B cells were capable of phosphorylating STAT3 in response to IL-21 in sIgAD compared to HC, whereas IL-10 induced STAT3 activation was normal in sIgAD B cells (Figure 2A). However, such an IL-21 driven STAT3 activation defect was not found in sIgAD T-cells. As depicted in Figure 6, in a visual manner, this defect was only seen for STAT3 but not all the other T-cell dependent and T-cell independent intracellular signaling pathways linked to IgA induction tested (Figures 3–5). The figure depicts the hypothesized model of sIgAD at the mucosal surface. The size of the B cell is exaggerated to draw forth the intracellular focus of the paper.

## Discussions

In this study, we identify defective STAT3 phosphorylation responses to IL-21 in sIgAD B cells. We were able to do so after mapping out the complex and heterogeneous intracellular signaling cascades following various stimuli known to be important in IgA induction. We both look at T-cell dependent cytokines and T-cell independent TLR9 responses. Delineating the intracellular signaling pathways under T- cell dependent and independent stimulatory conditions linked to IgA induction revealed that IgA induction in B cells may be driven through various pathways. Both IL-21 and IL-10 have had a strong link to sIgAD (5), and it was of special interest that both induced the strongest STAT3 phosphorylation of tested stimuli. But looking at sIgAD individuals we only identified a defect in IL-21 driven STAT3 phosphorylation. This potential defect may have several underlying causes. The defect may be linked to some narrowly defined effector B cell sub-population, since recently we have for example identified a defect involving transitional B cells in sIgAD (7). But the defect may also reflect a secondary dysregulation due to abnormal reactions in IgAD to heterogeneous stimulations, with common gamma chain cytokines, IL-10 or TRL9, needed for a correct IgA class switching (5, 6, 9–12).

IL-21 has been claimed to be the most promising curative target in sIgAD (3). A role that could be supported by our observations. The differences in responsiveness seen in sIgAD could be hypothesized to be downstream of the IL21 receptor but the normal responses seen with other stimuli make this highly unlikely. We rather see a cytokine specific response pattern for IL-21 (7), highlighting the potential role for IL-21 in antibody deficiencies. In addition, given the normal response to all IL-10 driven down-stream signaling events in our study it is not surprising that IL-10 is not capable to normalize IgA production in sIgAD (7). IL-21 is furthermore the cytokine that is most strongly linked with germinal center maturation where class switched B cells emerge, which have recently been shown to be abrogated in numbers and percentage in sIgAD (7). While IL-21 is known to be secreted by T follicular helper cells (TFh) in germinal center reactions, this lymphocytic sub-population does not seem to be the culprit in the pathogenesis of sIgAD since these are normal in number and function in sIgAD (7). However, it is conceivable that a subtler defect might exist in *in vivo* germinal center settings not detected in our model. This could also be linked to TLR9 defects seen prior (7) since the co-stimulation of CpG and IL-21 has been shown to enhance the STAT3 phosphorylation in humn B cells (23).

The heterogeneity in responses of sIgAD B cells to common gamma chain cytokines has been of interest given their overlapping signaling pathways (14). Common gamma chain cytokines have in common that their respective cell surface receptors all contain the common gamma amino acid chain (CD132) and they utilize intracellular signaling in similar ways. The main signaling pathway responsible in relaying signals of these cytokines is the JAK/STAT signaling pathway (13). JAK proteins phosphorylate different STAT proteins which then affect transcription of target genes (5, 9, 10, 12). The heterogeneous signaling responses seen in this study do though underline differences in the function of these cytokines. Furthermore, in healthy individuals a novel synergetic effect was noted for IL-4 and IL-10 on STAT5 phosphorylation in T-cells and B cells pointing toward multiple stimuli scenarios leading to different modulations. These responses did not occur when the cytokines were used separately. Something that could be affecting the fine tuning through multiple inhibitor approaches in clinical settings. The results do furthermore reflect on the importance of analyzing complex arrays of intracellular signaling events in various sub-populations of lymphocytes. Whereas, through several simultaneous intracellular pathways, phosphoflow offers the addition of getting a pattern of many pathways at once for separate cell populations with minimal chance of affecting activation status of cells via isolation.

The fact that a signaling defect is only seen after T cell dependent but not T cells independent mimicking stimulations is of interest given the stronger induction of IgA after CpG than T cell dependent stimulation in both the HCs and sIgAD cohorts (7). Thus, in another and related PAD, CVID, a defect has recently been shown after TLR9 stimulation in STAT3 signalization (19). Even though our observations described here do not demonstrate a defect involving the TLR9 driven signaling pathway, it was interesting that a defect involving STAT3 activation is shared for both conditions. However, in our model no meaningful STAT3 signaling was noted following CPG-TLR9 driven activation, even in HCs. In addition, TLR9 activation has been predominantly linked to the IRAK4, MYD88, and ERK, supported by our findings and described here in defined lymphocyte sub-populations (24).

The limitations of the study like with all studies estimating intracellular phosphorylation proteins are the phospho epitopes stained for. Even though the phosphorylation sites on tyrosine fragments stained for are the primary site for signal transduction other phosphorylation patterns do exist, for example serine phosphorylation of STATs (25). With regard to the activation of B cells in sIgAD it could be argued that the phosphorylation of plasma blasts in the periphery is not detected, because of their paucity and low expression of CD20, but since it has been recently shown that these are not present in peripheral blood of sIgAD individuals (7) and may not be most sensitive to the chosen stimuli targeting naïve and memory B cells. To fully elucidate the potential involvement of other lymphocytic sub-populations confined to the primary and/or secondary lymphoid organs a more detailed analyzes on these organs would be needed. However, these were not accessible to us at the time of the study due to ethical considerations.

It could be argued that basal phosphorylation could influence the analysis of differences between deficient and non-deficient individuals. Especially since it has been shown that the constitutive (basal) levels of phosphorylated kinases can vary in various pathological states we compared the phosphorylation of unstimulated samples in sIgAD compared to HCs (22). However, no noticeable difference was found between the two groups when evaluating basal phosphorylation state for any of the intracellular pathways evaluated in both HC and sIgAD in our study.

The above findings further strengthen a potential role for IL-21 as a therapeutic target in sIgAD, that could be monitorable through intracellular signaling events of STAT3 (5). Further research is needed though before administrating IL-21 or other cytokines to sIgAD individuals to revert the defect since special care should be taken with regards to defects in longevity of plasma cells in IgAD (7), as the amount of autoantibodies (26–28) and their potential inducibility in PADs could be enhanced with such exogenous stimuli (29). Whereas, the cytokine abrogating effects of biologicals and novel JAK/STAT inhibitors might have a role in the treatment of sIgAD or other hypogammaglobinaemia's with concomitant autoimmunity. This might be especially true in circumstances with sIgAD in connection to severe forms of atopic and/or autoimmune manifestations given their potential and emerging heterogeneous functional clinical responses (30).

In conclusion, our findings clarify the pathways activated by different stimuli linked to IgA induction. They furthermore shed light on the prior hypothesized immunotherapeutic role of IL-21 to revert the paucity in immunoglobulin subtype production in PADs by describing a defect in B cell phosphorylation after IL-21 in pSTAT3 in IgAD. Further research is still needed to develop a therapeutic approach based on these results in sIgAD and other related diseases.

## Data Availability

The datasets generated for this study are available on request to the corresponding author.

## Author Contributions

AL, FT, HE, and BRL conceived, designed the experiments, and wrote the paper. AL and FT performed the experiments. AL, FT, and BRL analyzed the data.

### Conflict of Interest Statement

The authors declare that the research was conducted in the absence of any commercial or financial relationships that could be construed as a potential conflict of interest.

## Supplementary Material

Supplement Figure 1 Basal phosphorylation in B and T cells from sIgAD and HCs. The figure shows % of phosphorylated proteins as measured *ex vivo* in PBMCs. **(A)** B cells (CD20+) and **(B)** T cells have a similar basal phosphorylation of STAT3, STAT5, STAT6, and expression of ERK1/2 in sIgAD individuals compared to HCs. The variation does not differ between measured proteins other that pSTAT3 in T cells (Figure 1B). Significance was calculated in relation to the control group.

Supplement Figure 2 Validation of staining for STAT1, STAT3, STAT5, STAT6 signaling pathways. PBMCs were isolated from a healthy individual then either left unstimulated or stimulated with IFN-γ, IL-21, IL-2, or IL-4 for 15 min then fixed, permeabilized and stained for phosphorylated STAT1, STAT3, STAT5, STAT6 then analyzed with flow cytometer. Orange is a negative control sample unstained and unstimulated, blue is an isotype control sample stimulated, green is stained sample unstimulated, and gray is stained sample stimulated. Lymphocytes are displayed on the left and monocytes on the right.

Supplement Figure 3 Gating strategy for experiments. **(A)** Gating on single cells and CD3/CD20 positive cells. **(B)** gating of STAT1 in stimulation giving positive stimulation: IL-10, IL-10 + IL2, IL-10 + IL4, and IL-21 stimulation. Staining of an sIgAD individuals with IL-21 is shown in the box marked sIgAD. **(C)** Staining of pSTAT5 after IL-10, IL-10 + IL2, IL-10 + IL4 stimulation. **(D)** Stating of pSTAT6 after IL-4 and IL-10 + IL4 stimulation. **(E)** Staining of ERK after CpG stimulation.

## Abbreviations

CD40L, CD40 ligand: alternatively CD154
CVID: Common variable immunodeficiency
ERK: Extracellular regulated kinases
GMFI: Geometric mean of fluorescence intensity
HCs: Healthy controls
Ig: Immunoglobulin
IL: Interleukin
JAK: Janus kinase
PAD: Primary antibody deficiency
PBMC: Peripheral blood mononuclear cell
PBS: Phosphate buffer saline
RPM: Rotation per minute
sIgAD: Selective immunoglobulin A deficiency
STAT: Signal transducer and activator of transcription
Tfh: T follicular helper cell
TGF- β: Transforming growth factor β
TLRs: Toll-like receptors.
