# Supplementary figures and images for: Mapping of Signaling Pathways Linked to sIgAD Reveals Impaired IL-21 Driven STAT3 B-Cell Activation
Source: Front Immunol. 2019 Mar 18;10:403. doi: 10.3389/fimmu.2019.00403 (PMC6431630; doi:10.3389/fimmu.2019.00403)

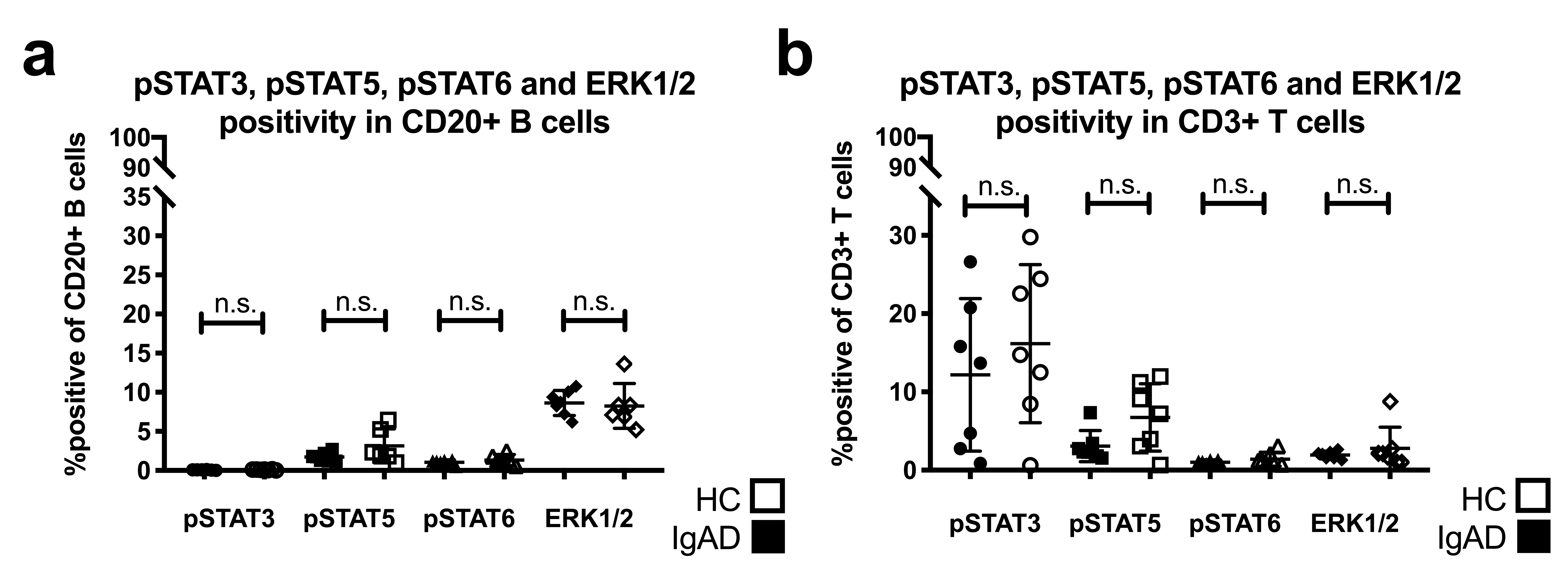

Supplement: Supplement Figure 1 — Basal phosphorylation in B and T cells from sIgAD and HCs. The figure shows % of phosphorylated proteins as measured ex vivo in PBMCs. (A) B cells (CD20+) and (B) T cells have a similar basal phosphorylation of STAT3, STAT5, STAT6, and expression of ERK1/2 in sIgAD individuals compared to HCs. The variation does not differ between measured proteins other that pSTAT3 in T cells (Figure 1B). Significance was calculated in relation to the control group. [file Image_1.TIFF]

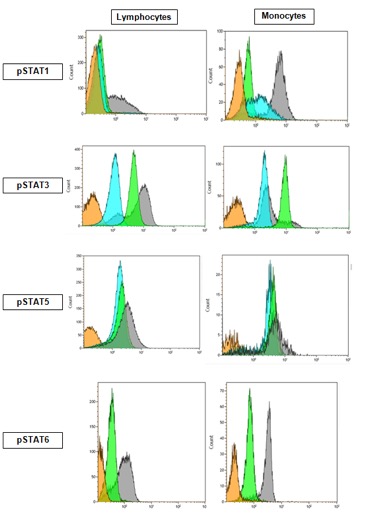

Supplement: Supplement Figure 2 — Validation of staining for STAT1, STAT3, STAT5, STAT6 signaling pathways. PBMCs were isolated from a healthy individual then either left unstimulated or stimulated with IFN-γ, IL-21, IL-2, or IL-4 for 15 min then fixed, permeabilized and stained for phosphorylated STAT1, STAT3, STAT5, STAT6 then analyzed with flow cytometer. Orange is a negative control sample unstained and unstimulated, blue is an isotype control sample stimulated, green is stained sample unstimulated, and gray is stained sample stimulated. Lymphocytes are displayed on the left and monocytes on the right. [file Image_2.JPEG]
